# A Thermo-Catalytic Pyrolysis of Polystyrene Waste Review: A Systematic, Statistical, and Bibliometric Approach

**DOI:** 10.3390/polym15061582

**Published:** 2023-03-22

**Authors:** Arantxa M. Gonzalez-Aguilar, Vicente Pérez-García, José M. Riesco-Ávila

**Affiliations:** Mechanical Engineering Department, Engineering Division, Campus Irapuato-Salamanca, University of Guanajuato, Salamanca Gto. 36885, Mexico; am.gonzalez.aguilar@ugto.mx (A.M.G.-A.); v.perez@ugto.mx (V.P.-G.)

**Keywords:** polystyrene, pyrolysis, bibliometric, statistical

## Abstract

Global polystyrene (PS) production has been influenced by the lightness and heat resistance this material offers in different applications, such as construction and packaging. However, population growth and the lack of PS recycling lead to a large waste generation, affecting the environment. Pyrolysis has been recognized as an effective recycling method, converting PS waste into valuable products in the chemical industry. The present work addresses a systematic, bibliometric, and statistical analysis of results carried out from 2015 to 2022, making an extensive critique of the most influential operation parameters in the thermo-catalytic pyrolysis of PS and its waste. The systematic study showed that the conversion of PS into a liquid with high aromatic content (84.75% of styrene) can be achieved by pyrolysis. Discussion of PS as fuel is described compared to commercial fuels. In addition, PS favors the production of liquid fuel when subjected to co-pyrolysis with biomass, improving its properties such as viscosity and energy content. A statistical analysis of the data compilation was also discussed, evaluating the influence of temperature, reactor design, and catalysts on product yield.

## 1. Introduction

Plastic is a non-biodegradable material referred to as the “material of every application” [1]. The demand for plastics continues to increase owing to their inherent properties such as light weight, low cost, flexibility, durability, resistance to erosion, versatility, moldability, and ease of production and maintenance [2,3,4,5]. Currently, plastic products are an indispensable part of people’s daily lives. They are applied in various industries, such as construction, healthcare, electronic and electrical components, agriculture, automotive, aviation, textiles, household, and packaging [5,6,7,8,9].

Since 1950, global plastic production has grown at a compound annual growth rate of 8.40%; the annual plastic production was 390 million tons in 2021, and plastic production is estimated to reach 500 million tons in 2025 [10,11]. Worldwide, the plastic demand is increasing annually by 4% [12]. Polystyrene (PS) is heat resistant and light in weight; it has good strength and durability, making this polymer suitable for various applications such as food packaging, beverages, household appliances, the automotive field, and insulating systems for the construction industry [13,14,15,16,17]. PS accounts for around ten wt.% of the total plastic waste produced annually in the last ten years [18].

The plastic industry is ubiquitous worldwide, and the generation of “plastic waste” is a consequence of industrial development steadily increasing to the point of being considered a high-impact pollutant and causing severe environmental problems [19,20,21].

Recycling and reusing plastic waste is vital for various reasons, and the most important of all is the conservation of natural resources and the reduction in environmental pollution [19]. Plastic recycling provides a “waste-to-value” strategy to utilize used plastics as a resource to produce valuable commodities, which simultaneously contributes to plastic waste management and sustainable development [22]. In recent years, increased attention has been paid to recycling synthetic polymer waste, solving pollution problems, and reusing cheap and abundant waste products [13].

Plastic recycling technologies began to develop in the 1970s, and there have been many advances since then. Primary and secondary recycling involves the mechanical reprocessing of used materials into products with little or no effects on their physical properties. It can also be considered physical recycling; sorting, extrusion, segregation, grinding, and melt-processing are the most commonly used methods in the industry [23,24]. Mechanical recycling is often referred to as the most promising recycling technology in energy consumption; nevertheless, it prefers pure single-polymer streams, which are difficult to obtain due to the mixture of several plastics [25]. Recycling plastics has been proven to be a problematic, costly technique due to various constraints such as water contamination, and good sorting is needed before recycling [26].

On the other hand, tertiary recycling involves valuable chemical intermediate recovery (depolymerization, solvolysis, pyrolysis, gasification, hydrocracking, and others) [27,28,29,30]. Nowadays, chemical or feedstock recycling is attracting much attention, as it is environmentally friendly [31]. Chemical recycling overcomes the quality issue of thermoplastic recycling by enabling the production of virgin materials such as monomers, oligomers, and higher hydrocarbons from chemically-recycled feedstock [27,32]. Finally, quaternary recycling involves energy recovery (incineration) [20]. Solutions such as landfilling and incineration have many drawbacks (high energy cost, loss of carbon, and others) and thus, low economic efficiency [33]. In addition, these strategies are unaffordable for developing countries because of their global warming mandate [13,34]. Incineration for energy recovery can cause environmental issues by emitting dioxins, NO_x_, SO_x_, heavy metal oxides, or polycyclic aromatic hydrocarbons [35,36].

The study of this review article aims to provide readers with a synthesis of the work on PS thermo-catalytic pyrolysis through an extensive literature review of recent investigations. The bibliometric analysis will identify the state of the art of PS pyrolysis by analyzing mainly the areas of study where this type of process has been studied; moreover, it will show the potential for further development and research in countries related or, in contrast, will open an area of opportunity in those countries where PS pyrolysis has not been investigated. Finally, another critical point addressed in this review article is the statistical analysis of the data collected to show descriptively and graphically the operating parameters that have most influenced the polystyrene pyrolysis process over time.

## 2. Pyrolysis as an Alternative Plastic Waste Recycling Route

Plastic polymers are, in principle, the derivatives of fossil fuels and petrochemicals.

The long chains of polymers have several thousand repeating units of monomers, which makes plastic durable [15]. Remarkably, the high degradation stability and low density of PS cause significant problems when disposed of in a landfill; thus, processing PS waste is an important issue. Since non-renewable hydrocarbon feeds are used for the production of polymers, the chemical processing of polymer waste to produce fuels or petrochemical feedstocks is more favorable compared to other recycling methods [33,37]. Additionally, chemical recycling of plastic waste to base chemicals represents a promising recycling route since it is expected to be more robust towards impure and contaminated plastic waste streams. In pyrolysis, plastic waste is decomposed thermal or catalytic chemically at elevated temperatures (300 to 900 °C) without oxygen to convert polymers into small molecules to obtain liquid, gas, and solid fraction [7,20,24,38].

Managing plastic wastes via pyrolysis is an up-and-coming valorization technique, as it permits the use of this waste, without previous processing, to obtain products with high added value [39,40]. The main applications of pyrolytic oil derived from plastic waste are fuels [41,42,43,44,45,46,47] or chemical feedstock [23,48,49,50,51] that can be used to manufacture virgin-quality polymers [5]. Pyrolysis is a flexible recycling method because the pyrolytic products can be modified by varying the reaction conditions [14,52]. The significant factors influencing the plastic pyrolysis product molecular distribution include plastic chemical composition, reaction temperature, residence time, heating rate, operation pressure, reactor type, and catalyst application [8,37,53,54,55,56,57].

## 3. Methodology

The present review article addresses a systematic, bibliometric, and statistical analysis of polystyrene’s thermal and catalytic pyrolysis (virgin, EPS, and HIPS). The database was filtered to the years from 2015 to 2022. The systematic analysis compiles information in summary tables and graphs, estimating parameters’ ranges. GetData 2.26 software (Sarov, Russia) was used for recollecting some data from graphics reported in the literature with a risk of bias of ±2%. On the other hand, the bibliometric study was conducted by the Web of Science (WoS) database. In addition, it was developed using the VOSviewer 1.6.18 software (Centre for Science and Technology Studies, Leiden University, The Netherlands) to obtain a networking visualization map of co-authorship and the leading countries in PS pyrolysis publications. Finally, the statistical analysis is based on a visual representation using box plots (25–75% quartiles), describing the mean (±95% confidence intervals) and outliers to compare the distributions and observe influential parameters in the yields of the products derived from PS pyrolysis. The statistical analysis was performed using the OriginPro 2022 software (Northampton, MA, USA). The authors of the present review have read and followed the PRISMA guidelines [58].

## 4. Polystyrene Characterization

Plastics are synthesized from organic polymers or long chains of carbon atoms in addition to hydrogen, oxygen, nitrogen, sulfur, and chlorine [15]. Moreover, thermoplastics are a family of plastics that can be melted when heated and hardened when cooled. These characteristics, which lend the material its name, are reversible. It can be reheated, reshaped, and frozen repeatedly [59].

Polystyrene is one of the polymers found in 1930, made through a polymerization process with additives. PS is made up of a long hydrocarbon chain with phenyl groups linked to alternating carbon atoms, and it is obtained from the liquid petrochemical and can be found in solid or foam states [14,15]. PS is identified by code 6 and has a chemical formula of (C_8_H_8_)_n_ and the International Union of Pure and Applied Chemistry (IUPAC) name of poly(1-phenylethene) [60]. There are different presentations of PS; among them, the following stand out: expanded polystyrene (EPS) and high-impact polystyrene (HIPS). PS is transparent but can be colored by colorants [14].

In contrast, a typical EPS is made up of 98% air and 2% PS [61]; EPS is produced by the expansion of styrene pellets [62]; the process is known as pre-expansion [13,62]. These pre-expanded beads are kept in mesh storage silos for around 24 h to diffuse air into the beads [63]. Finally, HIPS is the co-polymer of polystyrene and butadiene rubber with improved toughness compared to PS [64]. Table 1 compiles the chemical properties of virgin PS and PS, EPS, and HIPS wastes found in the literature. 

PS represents a high carbon content plastic; virgin PS can contain at least 91.23 wt.%, and it is observed that when it is discarded, the carbon content decreases. However, HIPS waste is the type with the least amount of carbon, possibly because additives accompany this material. The additives in HIPS also increase the oxygen and ash content, HIPS being the material with up to 17.84 and 7.60 wt.%, respectively. In addition, PS material is composed of volatile matter with a maximum of 99.90 wt.% when it is virgin, decreasing to 88.90 wt.% when discarded. The high amount of volatile matter makes PS an excellent candidate for chemical recycling, achieving high conversion yields.

All PS types are difficult to be degraded by nature, producing a problem for the environment [71,72].

## 5. Bibliometric Analysis of PS Pyrolysis

**Figure 1:**
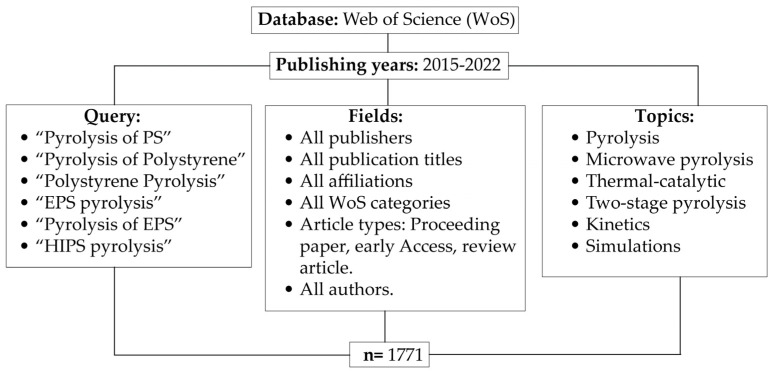
Criteria of bibliometric analysis of PS pyrolysis.

The study of bibliometric networks, such as co-authorship, bibliographic coupling, co-citation, and publishing countries, has received considerable attention in building control research [73,74]. This type of analysis helps to know the state of the art of a given topic and its potential for further development and research. Data analysis research covers different fields such as materials, education, economy, medicine, environment, energy, and others [75,76,77,78,79,80,81,82,83,84,85,86,87,88,89,90,91]. Armenise et al. [2] developed a bibliometric analysis of the pyrolysis of different plastic wastes. They demonstrated four research hotspots in plastic pyrolysis research: a thermal and kinetic study on plastic degradation, catalysis on the pyrolysis performance, co-pyrolysis of biomass and plastics, and the process parameter’s performance. The present review article addresses a bibliometric approach of thermo-catalytic pyrolysis exclusively from polystyrene. Figure 1 describes the following criteria for the bibliometric study.

From the WoS database and the specified query, 1771 records were found. Of the total, 1200 were screened, and 571 were excluded by humans. However, 63 studies were included in the present review article described in tables and graphs, in addition to the statistical analysis. The reasons for excluding publications were: general duplicate information (22) including recycling or pyrolysis reviews with general information; simulation/modeling (16); different parameters or types of pyrolysis (311), these included pyrolysis of other feedstocks, advanced pyrolysis methods, studies of the influence of other operating parameters not included in the present review; other techniques relevant to polystyrene (63); and articles with no relevance to PS pyrolysis (49).

**Figure 2:**
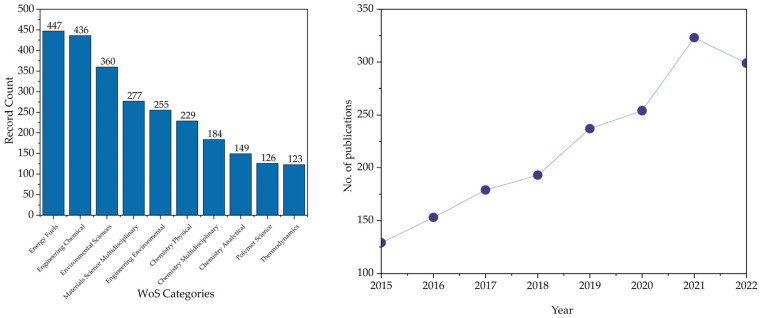
The top ten publishing categories and publications per year.

Figure 2 shows the top ten categories into which the PS pyrolysis topic is classified and the trend of publications from 2015 to 2022. It is observed that the main categories are associated with the approaches of PS pyrolysis: obtaining alternative fuels, producing high-value products in the chemical industry, and the involvement of this process with existing environmental issues. Energy Fuels has 447 records, followed by Engineering Chemical and Environmental Sciences with 436 and 360 publications, respectively.

In terms of publishing years, there is a significant increment of 129 to 179 publications between 2015 to 2017, adding approximately 25 extra records per year. There was a slight decrement in 2018, with only 14 additional documents representing 193 publications. Despite this diminution, the following years show another increment of up to 323 papers. In 2022 there is a diminution of 24 records compared to 2021.

**Figure 3:**
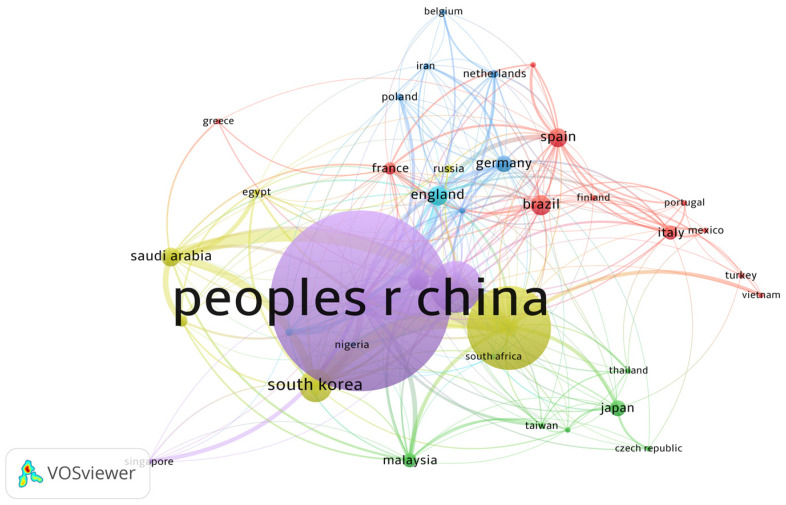
Leading countries in publications of PS pyrolysis.

Figure 3 indicates the countries that publish the most about PS pyrolysis. The following criteria were with full counting method, ten maximum number of countries per document, ten minimum number of publications of a country, and ten minimum number of citations. The analysis showed 91 countries involved, of which only 37 meet the based criteria. It is observed that 37 items are organized in 6 clusters. The leading publishing countries are China, with 641 publications and India and USA following with 299; Mexico only showed 18 publications.

**Figure 4:**
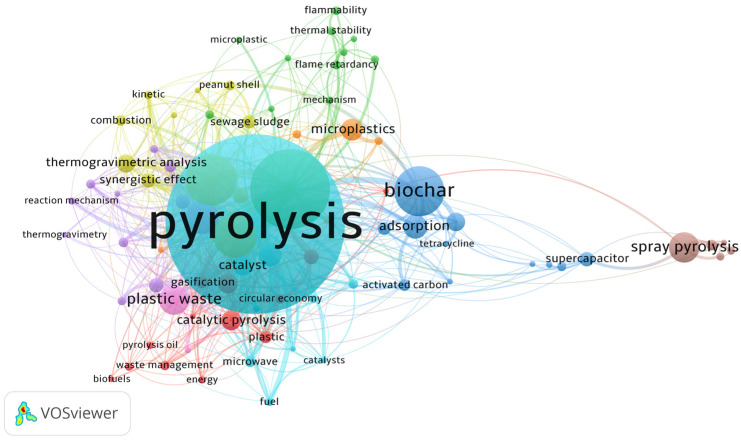
Co-occurrence of author keywords from PS thermo-catalytic pyrolysis research.

Keywords represent the essential content of a research paper. Figure 4 shows the results from a co-occurrence author keywords analysis from thermo-catalytic pyrolysis of PS intending to provide the approaches of the latest published articles. The study was executed with a full counting method and ten minimum keyword occurrences. Of 4247 keywords resulted, only 81 met the threshold grouped in 9 clusters. The results indicated that the keyword “Pyrolysis” is the central node and has 286 occurrences with 378 link strengths. Moreover, “Polystyrene” (128), “Co-pyrolysis” (83), “Biomass” (69), “Kinetics” (53), and “plastic waste” (48) are the following author keywords with the highest number of occurrences.

Based mainly on the results of the most important topics related to PS pyrolysis through bibliometric analysis, the present article review made a systematic and statistical analysis, and the results are discussed below.

## 6. Statistical Analysis of PS Pyrolysis

The interpretation of numerical data on a particular topic is facilitated by statistical analysis, making evident the distribution, normality, means, and outliers of a data set. A statistical analysis of the thermo-catalytic pyrolysis of PS can help to identify those parameters that have a significant influence on the yields or selectivity of the products. Therefore, in the present review article, data collected from the literature are statistically processed to reach the conclusions these data provide.

### 6.1. Thermal Degradation through TGA Analysis

Thermogravimetric analysis (TGA) determines the quantity and the frequency of the weight variation of the samples against temperature and time in a controlled atmosphere [92]. TGA is used to study the degradation behavior of polymeric materials, including homopolymers, copolymers, and others [93]. In addition, TGA helps determine degradation trends of operation parameters of the pyrolysis process, such as temperature, heating, oxygen absorption rates, and others [64]. In the literature, different studies on thermal or catalytic pyrolysis of PS have been developed; in these publications, the authors reported as a first step a thermogravimetric analysis of their samples to determine the optimum reaction conditions in the pyrolysis process and to develop their design of experiments.

Fuentes et al. [94] reported the initial, final, and maximum temperatures of two types of PS, purchased and waste. Their results showed slight differences in the degradation temperatures. It was observed that the purchased and the waste had a weight loss of 98.50 and 95.60% at a final temperature of 452 and 463 °C, respectively. Despite these differences, the maximum degradation temperature for both samples was about 420 °C.

**Figure 5:**
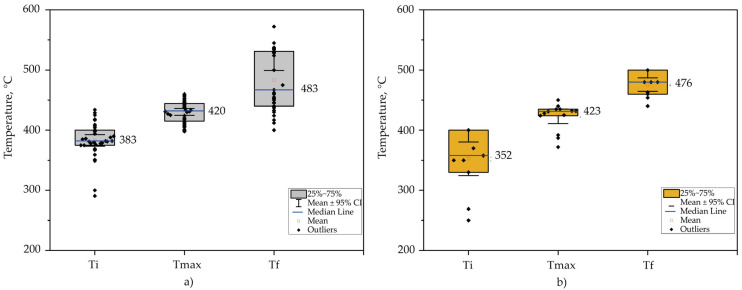
Degradation temperatures by TGA [initial (Ti), maximum (Tmax), and final (Tf)]: (**a**) virgin PS; (**b**) PS waste.

Figure 5 visualizes box plots of data collected from the literature to visualize the distribution of virgin PS [94,95,96,97,98,99,100,101] and PS waste [54,55,67,94,96,102,103,104] degradation temperatures (initial, final, and maximum). The results contemplate data from TGA analysis of samples without catalyst in a nitrogen environment with a flow rate range of 10 to 100 mL min^−1^ and a heating rate of 3 to 100 °C min^−1^.

It can be observed in TGA results that the mean initial temperature of the PS waste is about 31 °C lower compared to virgin PS; an influential factor may be the impurities contained in the sample when the waste has not been pre-treated, such as in food. However, both maximum and final temperatures have similar behavior.

Recently, Van der Westhuizen et al. [67] evaluated the effect of feedstock contamination on PS thermal pyrolysis performance. Their results showed that contamination of polystyrene makes a difference in the liquid yield, reducing the yield by up to 6.40%.

### 6.2. Thermal Pyrolysis of PS

Thermal pyrolysis is the simplest form of chemical recycling in which carbon-carbon bonds are broken by applying heat [101]. A simple process can recover valuable chemicals; an example is the study by Lu et al. [95]. In their experiments, pyrolysis was performed under an inert nitrogen atmosphere and heating of 5 °C min^−1^ until reaching a temperature of 420 °C for 2 h. The investigation yielded 76.24, 13.01, and 10.75% of liquid, solid, and gas, respectively. The results stand out because they achieved a single styrene component in their liquid sample, reaching a 73% yield. The present review article analyzes the influence of temperature and reactor design on the thermal pyrolysis of PS by collecting data from the literature.

#### 6.2.1. Temperature

Temperature controls the cracking reaction in the polymer chain, which is why it is considered one of the most influential parameters in the pyrolysis process [7,53]. Studies report that, in the pyrolysis process, the production of a liquid with long hydrocarbon chains is improved at low temperatures. In contrast, the liquid yield decreases at high temperatures, and gas production improves. Furthermore, high temperatures lead to secondary reactions within the reactor, which reduce the obtaining of solid products [14].

A study of the influence of temperature on the thermal pyrolysis of PS waste is that of Verma et al. [70]. They evaluated the process at temperatures from 400 to 700 °C with 50 °C intervals and a heating rate of 15 °C min^−1^. Their results showed that the pyrolytic liquid yield increases with increasing temperatures up to 650 °C. However, the liquid yield decreases at temperatures higher than 650 °C. Moreover, the same behavior was obtained even when a catalyst was used.

**Figure 6:**
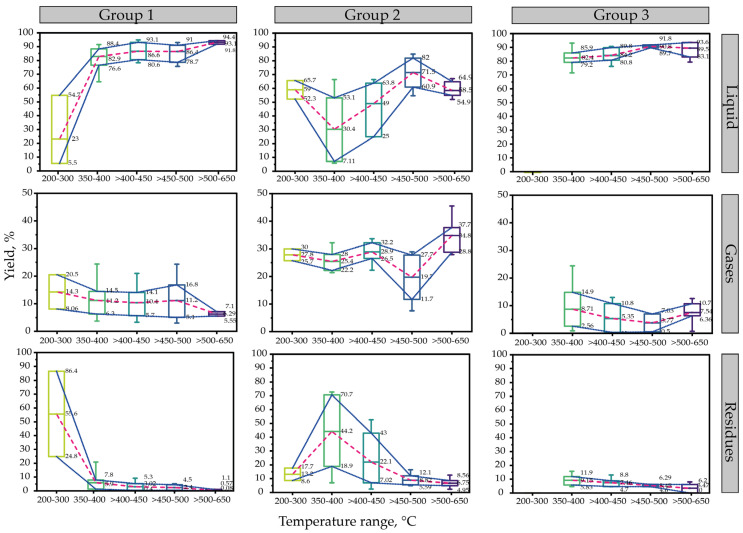
Influence of temperature on products yields in PS thermal pyrolysis.

Figure 6 shows the statistical analysis of the influence of the reaction temperature on the liquid yield in the thermal pyrolysis of polystyrene grouped into three groups of reactor design. Group one corresponds to batch, semi-batch, and batch reactors with agitation [16,18,55,64,70,101,102,104,105,106,107], while the second group belongs to the studies evaluated in fixed-type reactors [108,109]. Finally, group three corresponds to laboratory-scale reactors such as the Pyrex type or those reported as “Steel pyrolizers” [67,95,96,110,111]. In this case study, the influence of other operating parameters, such as residence time, heating rate, or type of raw material, was not considered.

In terms of liquid yield, there is a tendency to increase from 350 °C to temperatures close to 500 °C in all groups. In addition, there are no significant differences when increasing up to 650 °C. Furthermore, there is a decrease of up to 20% on fixed reactors when the temperature is raised to more than 500 °C. Additionally, except for fixed reactors, it is possible to obtain 80 to 90% liquid yields at temperatures from 350 to 500 °C. The low results reported within the range of 350 to 400 °C in group two correspond, among them, to those experimented on by Abdullah et al. [109]. The highest product obtained in their experiments was wax, obtaining up to 72.72% at temperatures of 350 °C and a heating rate of 30 °C min^−1^, approximately. In contrast, the highest yield obtained in these same variables was that of Sogancioglu et al. [108], who obtained up to 66.45% of oil at 400 °C with the difference that it was at a low heating rate of 5 °C min^−1^.

On the other hand, the distribution of gas yield in all groups is maintained at 10 to 40% for any temperature range. Finally, the results agree with Maafa [14], who stated minimal production of a solid fraction at elevated temperatures.

#### 6.2.2. Reactor Types

Reactor design is an essential parameter in the PS pyrolysis process since it affects how the reaction develops by influencing the method of heating, reactant mixing, residence time, and heat transfer.

Batch reactors are those where the system does not allow the flow of reactants or the exit of the products while the reaction is taking place; this allows the reactants to remain inside the reactor for a longer time, achieving a high conversion efficiency [112]. Moreover, these reactors are recognized for their simple design and ease of control of the operating parameters involved. Nevertheless, the main disadvantages of batch reactors are the requirements to refill feedstock and, in addition, that it is impossible to use them at a high production scale.

Unlike a batch reactor, in semi-batch reactors, it is possible to feed reactants and collect the resulting products simultaneously. However, coinciding with the disadvantage of the batch type, they are suitable for smaller-scale production. Finally, fixed-bed reactors are categorized as simple in design and are generally used as secondary reactors [113]. Yet, a two-stage process is not considered economically viable since the products obtained are very similar to single-stage processes [14].

Different types of reactors have been studied in the pyrolysis of PS to obtain products of interest in the chemical or petrochemical industry. The most used reactors are batch [18,55,101,105,106], semi-batch [16,64,101,102,104], fixed bed [108,109], and laboratory scale, which include basic systems such as a simple steel tube or using Pyrex material [67,95,103,107,114,115]. Lopez et al. [38] reported that reactor design is vital because an inadequate design leads to undesired reaction conditions, decreasing the quality of the products and promoting the formation of undesired by-products, such as solid residues or tar. Therefore, an optimal design must ensure a high heat transfer rate for rapid heating of the polymer and reliable temperature control to avoid operating problems due to the nature of the plastic melting process.

**Figure 7:**
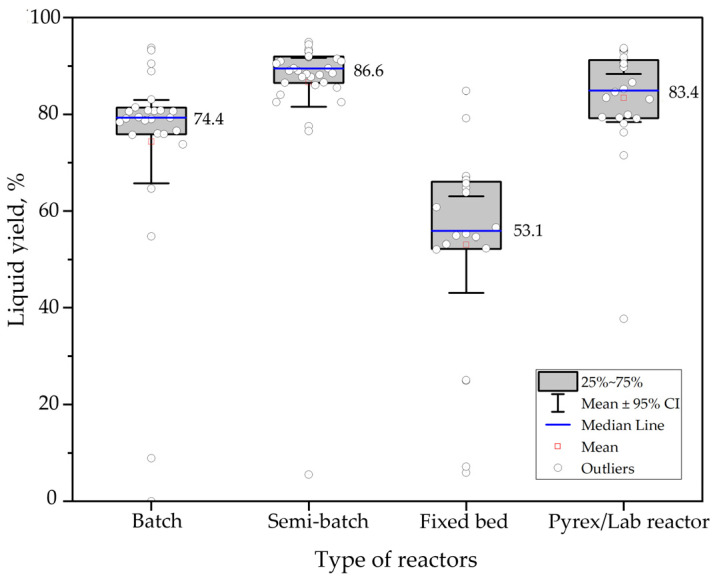
Influence of reactor types on liquid yield in PS thermal pyrolysis.

Figure 7 indicates the influence on the liquid yield of reactors used in PS thermal pyrolysis. The box plots show the data’s distribution organized by reactor type. In this case, the effect of temperature, residence time, heating rate, or feedstock was not considered. Experiments of heating rates from 4 to 200 °C min^−1^, residence times from 5 to 150 min, and minimum and maximum temperatures of 200 °C and 700 °C, respectively, were added to the analysis.

The results show that, on the mean, the semi-batch type reactors are the ones that obtain the highest liquid yield, remaining close to 86.60%, following laboratory-scale reactors with 83.40%. On the other hand, if fixed reactors are used, a mean of 53.10% of liquid hydrocarbon will be obtained. It is also observed that the distribution is dispersed to all reactors because no other parameters were contemplated in this case study; the data collected also vary by heating rate and temperatures, affecting product yields.

### 6.3. Catalytic Pyrolysis of PS

Thermo-catalytic pyrolysis has proven to be an alternative technology to reduce the impact of polymeric waste on the environment [13]. A catalyst is a substance that changes the performance of the chemical reaction without being altered in the process [24]. Using catalysts during a hydrocarbon degradation process can decrease the temperature and reaction time, increase the conversion rate, and promote a desired selectivity in the products [116]. These advantages make catalysts play an important role in waste pyrolysis processes [117].

The catalytic pyrolysis of PS has been less studied than other polyolefins; however, basic and acid catalysts have shown potential in the depolymerization of PS. Nevertheless, literature has shown differences using these catalyst types, such as the conversion efficiency of the products or their selectivity. An example is the study conducted by Inayat et al. [96], where they evaluated the catalytic pyrolysis of different PS wastes, experimenting with zeolite (HZSM-5) and MgO as acid and basic catalysts, respectively. Their results showed that implementing basic catalysts influenced the composition of the products slightly compared to those obtained by thermal pyrolysis. In conclusion, they indicated that the PS feedstock type affects the composition more than basic catalysts. Unlike acid catalysts, they significantly influenced the composition of the products.

Furthermore, in their experiments with zeolite, they added the operation mode as a factor (ex situ and in situ). The results indicated that mixing the PS waste with the zeolite increases the production of waxes. In contrast, zeolite promotes mono aromatics’ formation in the ex situ mode, such as benzene, toluene, and xylenes (BTX). The authors concluded that acid catalysts have the potential to transform PS-based materials into higher-value compounds.

#### Influence of Catalyst on Liquid Yield

**Figure 8:**
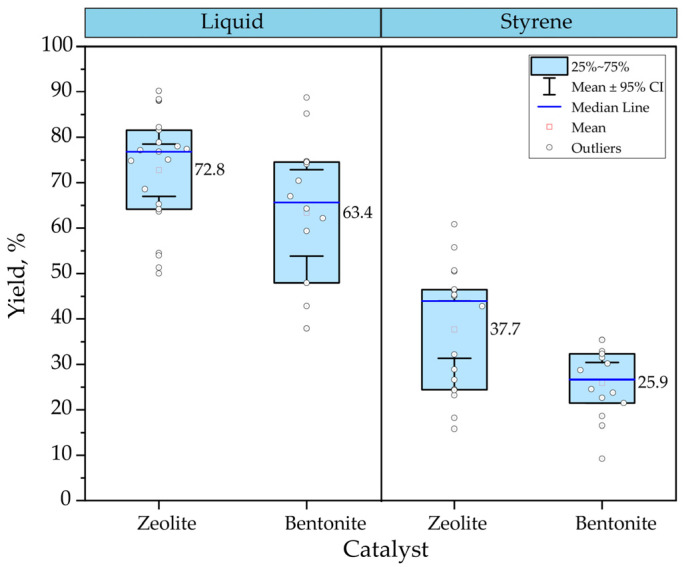
Comparison between zeolite and bentonite in the catalytic pyrolysis of PS.

Figure 8 compares two types of catalysts used in the catalytic pyrolysis of PS. The analysis involves data collected from experiments with different types of bentonite [64,70,96,111,118] and zeolites [72,118] and evaluates the influence on liquid hydrocarbon yield and styrene formation. In this case, the effect of another operating parameter was not contemplated; the analysis includes temperatures from 300 to 600 °C. Despite the wide scatter of the data collected, the results show that zeolite achieves the highest performance for both liquid yield and styrene formation. The yields mean between 37.70 and 25.90% liquid and styrene, respectively, with the use of bentonite; on the other hand, with zeolite, it is possible to reach, on mean, up to 72.80% liquid and 63.40% styrene.

Part of the data collected includes those reported by Dewangga et al. [118], in which they evaluated the behavior of bentonite and natural zeolite at different catalyst percentages, from 0 to 25%. Their results showed that, for both catalysts, the higher the catalyst percentage, the higher the liquid yield obtained. Specifically for zeolite, Rehan et al. [111] evaluated the performance of liquid yields with natural and synthetic zeolite in the catalytic pyrolysis of PS. Their results showed that using natural zeolites slightly favors the production of liquid hydrocarbon with 4% more than that obtained with synthetic zeolites. In contrast, gas production increases with synthetic zeolites, reaching approximately 10% more. Regarding styrene formation, the difference between the two types of zeolites is more notorious, obtaining about 60.80% and 15.80% for natural and synthetic zeolites.

**Figure 9:**
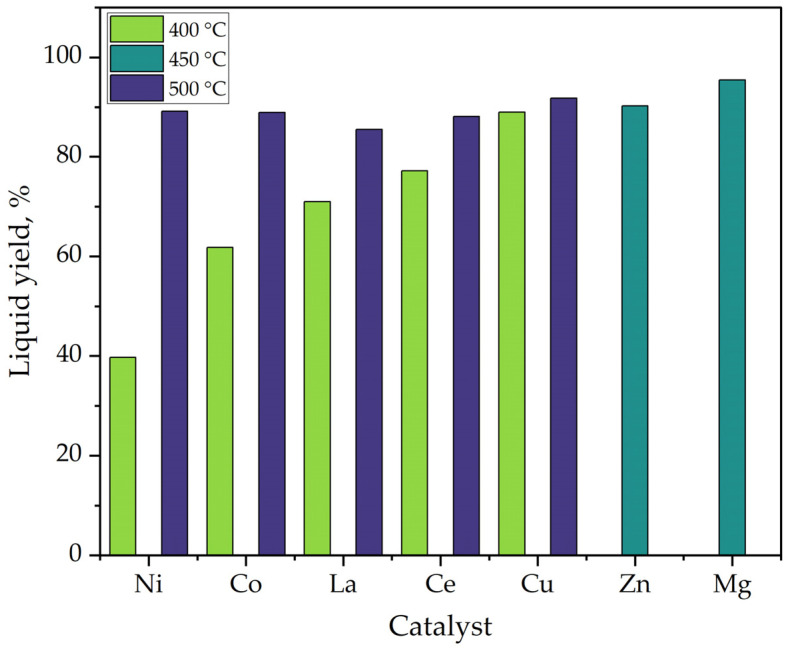
Liquid yield of different alumina-supported catalyst.

A comparison of the liquid yields obtained at temperatures from 400 to 500 °C with different metalsimpregnated on alumina (Al_2_O_3_)-supported catalysts is visualized in Figure 9. It is observed that all catalysts demonstrated high liquid conversion (85 to 90%) at temperatures from 450 to 500 °C, unlike Cu/Al_2_O_3_, where the temperature is reduced to 400 °C. Within the analysis are data reported by Adnan et al. [103], where they demonstrated that Zn-supported catalysts impregnated on Al_2_O_3_ resulted in better catalytic performance in PS pyrolysis over AC and Mmn supports. The yield of their liquid obtained was 90.20%, using a percentage of 20% Zn-Al_2_O_3_ at a temperature of 450 °C.

Within the case study, there is also the work completed by Çelikgöğüs [107]; their experiments were conducted under temperatures from 400 to 500 °C using different catalysts with Al_2_O_3_ support in a semi-batch reactor. Their results showed that using Cu/ϒ-Al_2_O_3_ enhanced the highest styrene conversion, up to 63.59%. Finally, the graphic also visualizes the performance of the work completed by Shah et al. [114]; their work aimed to evaluate magnesium-impregnated catalysts for the tertiary recycling of EPS waste in a Pyrex reactor. Their results determined that the maximum yield obtained was 95.47% with 15% Mg-Al_2_O_3_ at a temperature of 450 °C, achieving 56.20%, 13.10%, 8.93% of styrene, toluene, and ethylbenzene, respectively.

Additionally, catalysts are known for decreasing the temperature of a reaction. A highlighted work is by Aljabri et al. [119]; their objective was to evaluate the conversion of PS and its wastes into valuable products with high conversion at 250 °C with FeCo/Al_2_O_3_ bimetallic catalysts in a Parr stirred batch reactor. They achieved up to 91% liquid yield with a styrene monomer selectivity of up to 45 wt.% and ethylbenzene up to 55 wt.%.

### 6.4. Production of Mono-Aromatics from PS Pyrolysis

During pyrolysis, PS is cracked in its monomers (styrene) and dimers, trimers, and other compounds such as toluene, xylene isomers, methyl styrene, benzene, ethylbenzene, and others. The applications of these aromatic hydrocarbons include additives for blending with gasoline to promote high octane, paint solvents, precursors for synthesizing other chemical compounds, and the manufacture of resins and plasticizers [70,120,121]. Therefore, recovering those chemical compounds from plastic waste is essential to the recycling industry.

The amount of aromatic present in the pyrolytic oil depends on the type of reactor, reaction temperature, heating rate, and residence time; moreover, for catalytic pyrolysis, the yields also depend on the catalyst used. Table 2 summarizes the principal aromatics found in PS’s thermal and catalytic pyrolysis studies.

As shown in Table 2, in thermal pyrolysis the aromatic with the highest presence is styrene, followed by toluene, ethylbenzene, α-methyl styrene, benzene, and finally cumene. However, in catalytic pyrolysis, this order can vary depending on the catalyst used and the result sought. For example, Amjad et al. [102] used NiO as catalyst to decrease the concentration of styrene in the pyrolytic oil and to be able to use it as fuel. On the other hand, Rehan et al. [111] reported that by using zeolites as a catalyst, the benzene formation was promoted, achieving a maximum of 16.30%; moreover, they obtained the highest yield of cumene reported in the literature, about 8%. Rehan et al. [111] also used synthetic zeolite as catalyst and increased the formation of α−methyl styrene by up to 38.40%, compared with 9.50% obtained by Amjad et al. [102] via thermal pyrolysis. Finally, Aljabri et al. [18] reported that when using FeCu/alumina, the yield of ethylbenzene was improved, obtaining values within a range of 27 to 36%, approximately.

### 6.5. Production of Fuels from PS Pyrolysis

Obtaining alternative fuels from plastic waste has received significant attention because it can solve the problems of the final disposal of this type of waste. In addition, the growing demand for energy worldwide, the depletion of oil resources, and the high cost of petroleum-based fuels have led to the development of alternative fuels from different types of plastic waste [123,124].

Despite that, PS pyrolytic oil has significant aromatic content, and its application as a fuel is questionable; however, its main advantage is its high calorific content. Therefore, studies on producing a combustible derived from PS thermo-catalytic pyrolysis have been developed [52,54,67,125]. Nisar et al. [115] evaluated the obtaining of fuel from the pyrolysis of PS waste; the experiments were carried out in a salt bath reactor using copper oxide (CuO) as a catalyst. They reported that using the catalyst reduced the temperature and time of the process and increased the liquid produced during pyrolysis. Liquid properties were compared with some standard values from fossil fuels, and finally, they concluded that the liquid obtained had a great potential to be a substitute for commercial fuels.

**Figure 10:**
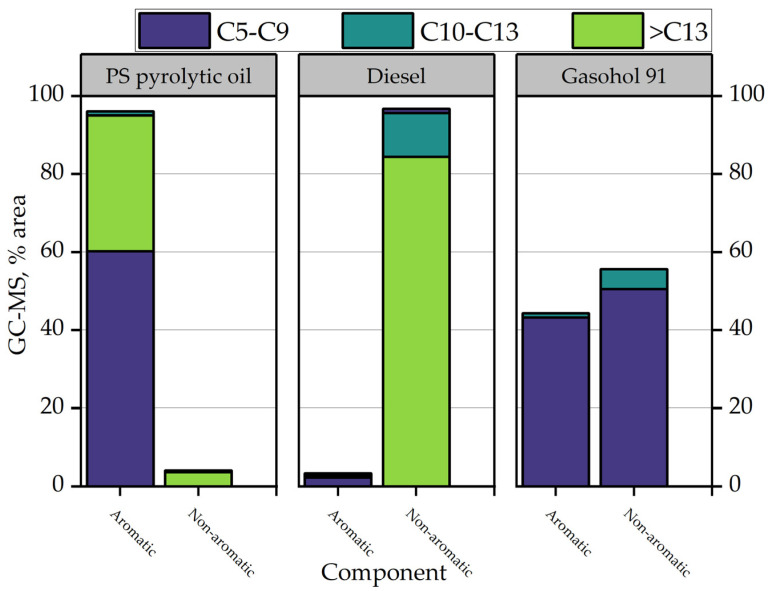
Carbon distribution of PS pyrolysis oil and commercial fuels.

Budsaereechai et al. [125] produced oil from plastic packaging waste in a bench-scale fixed pyrolysis batch reactor. Among their objectives, they studied the influence of heating rate on oil production; the results showed that the liquid yield decreases as the heating rate increases. Moreover, they concluded that oil derived from catalytic pyrolysis with commercial clay bentonite resulted in greater engine power, comparable engine temperature, and lower carbon monoxide (CO) and carbon dioxide (CO_2_) emissions than uncatalyzed oils and commercial fuel in the gasoline range. Figure 10 shows the distribution of carbons from C_5_ to higher C_13_ for the pyrolytic oil derived from this study, compared to diesel and gasohol 91. It is remarkable to identify the high content of aromatic components (C_5_–C_9_) in the PS oil derived from thermal pyrolysis with a value of 60%. In comparison, diesel only contains 2.18%, evidencing the significant difference between them. PS oil contains mainly short-chain (C_5_–C_9_) and long-chain (>C_13_) hydrocarbon chains, while medium-chain carbons characterize diesel.

Gasohol 91 is a fuel made from a mixture of gasoline and alcohol, usually ethanol or methanol, and is used in internal combustion engines. This fuel is commonly used in Thailand, and its composition is compared with the pyrolytic oil of PS [125]. The aromatic composition of gasohol 91 is relatively high, above 40%; a remarkable feature is that gasohol 91 contains almost entirely short-chain hydrocarbon (C_5_–C_9_), because it is a mixture of gasoline and alcohol. Budsaaereechai et al. [125] realized a similarity analysis by Fourier-transform infrared spectroscopy (FTIR) spectra, detailing different functional groups in the pyrolytic PS oil, diesel, and gasohol 91. The authors reported a low similarity between PS oil from thermal pyrolysis and diesel, about 17.90%, increasing to 20.75% when bentonite clay was added as a catalyst. In contrast, PS pyrolytic oil reached a percentage of similarity with gasohol 91 of 63.20 and 66.35% when thermal and catalytic pyrolysis were addressed, respectively.

In terms of fuel properties, density is a property used to know the approximate amount of fuel delivered by the injection systems for proper fuel combustion. On the other hand, kinematic viscosity plays an essential role in the atomization and penetration of the fuel jet [126]. Furthermore, fuel viscosity is a critical parameter because its value must be low enough to flow through the feed circuits without excessive pressure drops and, in some cases, such as diesel and fuel oil, high enough to meet specific lubricating requirements [127]. The flash point of a volatile material is the lowest temperature at which it can vaporize to form an ignitable mixture in the air. The flash point determines the volatility of liquid fuels, the amount of low boiling fraction present in liquid fuel, and explosion hazards [128].

Moreover, the pour point is the minimum temperature at which a liquid fuel loses its flow properties and is a critical property for the cold flow process. Finally, the energy content of a fuel is expressed by its calorific value, defined as the magnitude of the heat of reaction at constant pressure or volume and a standard temperature (usually 25 °C) for the complete combustion of a unit mass of fuel [129]. Table 3 summarizes the comparison of the described properties of commercial fuels with pyrolytic PS oils found in the literature.

The summary of fuel properties shows that the density of the liquid derived from PS at any temperature is relatively high, placing it out of the range of fuels such as gasoline, diesel, and kerosene. However, its value resembles marine application fuels, such as marine residual fuel (RMG-380) [67]. There is no trend in kinematic viscosity with pyrolysis temperatures; however, the values of 400, 500, and ~768 °C are within the established range for all commercial fuels compared. Gasoline and diesel must comply with a flash point above 42 °C, so pyrolytic oil, except for 550 °C, is compliant. Finally, one of the beneficial properties of PS pyrolytic oils is their high energy content. Due to the high aromatic content of PS oil, it is not recommended to be used directly as fuel. However, it is possible to blend it with other combustibles or biocombustibles to improve this property.

### 6.6. Co-Pyrolysis of PS and Biomass

Biomass is the biodegradable fraction of products, residues, and wastes of biological origin from agricultural activities, including substances of plant and animal origin, forestry and related industries, fishing, and aquaculture. Biomass is a biodegradable fraction of industrial and municipal waste of biological origin [134]. Biomass can produce alternative fuels, mitigating fossil fuel environmental and climate change impacts [135]. Moreover, co-pyrolysis refers to the process of thermo-catalytic pyrolysis in which different feedstocks are mixed, and the process is carried out. Several co-pyrolysis studies of biomass with waste plastics have been evaluated to enhance fuel production yields and improve fuel properties [136,137,138,139,140].

**Figure 11:**
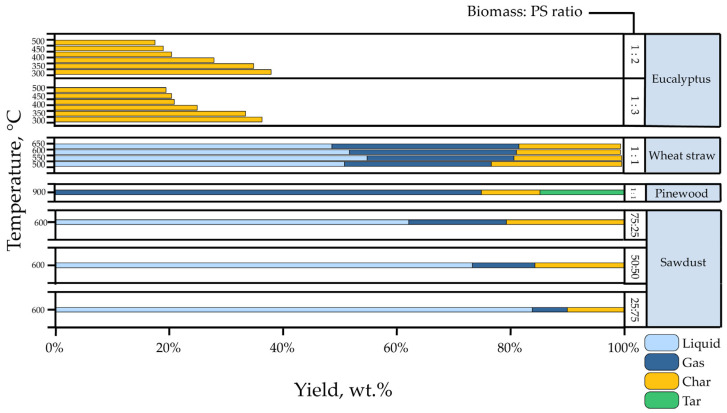
Co-pyrolysis of PS and various biomass types.

Figure 11 summarizes some of the product yields from the co-pyrolysis of biomass with PS. The production of char as potential fuel was carried out by Samal et al. [141]. Their experiments focused on evaluating the co-pyrolysis of PS waste and eucalyptus as biomass at varying temperatures from 300 to 550 °C, residence times from 90 to 150 min, and at different PS ratios (33 and 25%). Their results showed that char production decreases with increasing reaction temperature, obtaining a yield of 38.05 to 19.1% at temperatures of 300 and 550 °C, respectively, when 25% of PS is added. On the other hand, increasing the percentage of PS (33%) increases char production at temperatures above 450 °C.

Moreover, the results of Razzaq et al. [142] are also analyzed; they performed the co-pyrolysis of wheat straw and PS with a 1:1 ratio in a fixed-bed reactor. Their experiments reported the distribution of products obtained at temperatures ranging from 500 to 650 °C; liquid yield varied from 49 to 55 wt.%, with a maximum of 550 °C. Burra et al. [65] studied syngas production and co-processed pinewood as biomass and PS via pyrolysis and gasification at 900 °C. Their experiments were conducted in a fixed-bed reactor with unique sample-loading configurations; they obtained a yield of approximately 75%.

Finally, Stancin et al. [143] reported the co-pyrolysis of sawdust (oak, poplar, and fir wood) and PS waste to produce high-quality biofuels. Their experiments were carried out under a temperature of 600 °C and a residence time of one hour in a stainless-steel fixed bed reactor. Different blends were evaluated (PS/SD 25–75%, PS/SD 50–50%, and PS/SD 75–25%); the highest yield was achieved with 75% PS, obtaining 83.86% liquid yield. In contrast, by reducing the percentage to only 25%, the liquid conversion decreases to 62.33%. The authors concluded that the co-pyrolysis of biomass feedstock with different waste materials, especially plastics, might be a promising alternative for the sustainable usage of enhanced biofuels.

**Figure 12:**
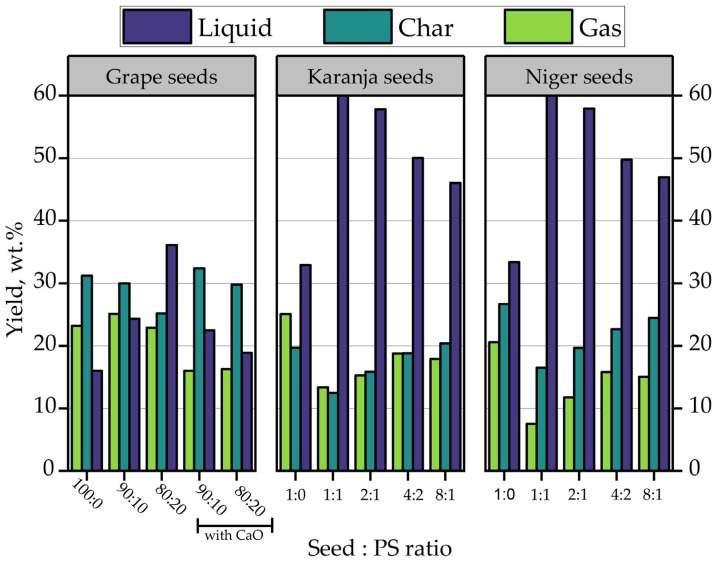
Co-pyrolysis of PS and various biomass seeds.

Studies of seeds with PS by co-pyrolysis were collected and compared; the results are shown in Figure 12. Veses et al. [144] experimented with thermo-catalytic co-pyrolysis of grape seeds (GS) and PS with CaO as a catalyst scaled up to an auger reactor plant. The agricultural wastes were blended with up to 20 wt.% of PS. The results indicated that the grape seed co-pyrolysis process yielded a low yield of 16 wt.% in the organic phase.

Nevertheless, with a co-pyrolysis with PS without a catalyst, the increment in PS percentage favored the liquid production, increasing from 24.30 wt.% to 36.10% with 10 and 20 wt.% of PS, respectively. On the other hand, using CaO within the co-pyrolysis did not favor liquid production compared to the thermal process, achieving at most 27.10 wt.% with 20 wt.% of PS. However, catalytic co-pyrolysis increased solid-phase CO_2_ formation by up to 3 wt.% in the presence of 10 wt.% of PS. Another study on co-pyrolysis of grape seeds and PS was performed by Muelas et al. [145], using Carmeuse as a catalyst, differentiating it from the previous one mentioned. Their experiments were performed at a GS: PS ratio of 80:20 in an auger reactor; the authors found that a significant improvement in the physicochemical properties of the bio-oils was obtained when using a catalyst with lower viscosity, density, and oxygen content. These benefits were most notable with Carmeuse as a catalyst due to the higher prevalence of aromatization and hydro-oxygenation reactions.

In addition, the authors evaluated the droplet combustion behaviors of the bio-oils produced. The tests revealed consistent micro-explosion occurrence, being higher for GS-PS oil; furthermore, the liquid yielded significantly higher burning rates during the initial heat-up phase due to its richer composition in volatile compounds such as styrene. Finally, the authors pointed out this fuel as the one with the best global combustion behavior over other bio-oils studied.

Another study using non-edible seeds such as Karanja and Niger with PS by co-pyrolysis in fuel production is reported [146]. Their research aimed to evaluate the increase in fuel quality by improving the calorific value and cold flow properties and reducing the viscosity of co-pyrolytic oil. The authors reported that adding PS in the process increased the conversion of seeds to liquid and significantly influenced the fuel properties. The highest liquid yield obtained for Karanja and Niger seeds was at a 1:1 ratio, reaching 60.11 and 61.31 wt.%, respectively. Additionally, increasing the percentage of seeds in the mixture decreases oil production. On the other hand, it is possible to reduce fuel viscosity up to 8.67% and 5.8% for Niger and Karanja seeds, respectively.

Regarding fuel energy content, another reason for blending PS pyrolytic oil with biomass or conducting co-pyrolysis of both feedstocks is the increase in the calorific value of the biofuels. Reshad et al. [147] evaluated the energy content behavior of biofuels derived from the pyrolysis of rubber seeds (RSC) with PS at different operating conditions. Their results showed that PS pyrolytic oil contained 42.10 MJ kg^−1^, and its high calorific value influenced the co-pyrolysis with the seeds. In the experiments with varied RSC: PS ratios, the results showed that the higher the amount of PS, the greater the increase in the calorific value of the biofuels, achieving up to 41.10 MJ kg^−1^. This same behavior can be observed in Table 4, except for Niger and Karanja seeds, where a seed:PS ratio of 2:1 maximizes the calorific value of the biofuels.

## 7. Conclusions

An extensive literature review (from 2015 to 2022) on PS and its waste thermo-catalytic pyrolysis resulted in a systematic, bibliometric, and statistical analysis. The bibliometrics indicated that keywords such as “co-pyrolysis”, “biomass”, “kinetics”, and “waste management” are the most studied in terms of PS pyrolysis. In addition, China, India, and the U.S.A. are the top three countries with the most publications on PS pyrolysis.

On the other hand, the systematic and statistical analysis showed that low temperatures are recommended if a pyrolytic liquid is required; in contrast, increasing temperatures enhance the production of the gaseous fraction. The PS pyrolytic liquid can contain in its composition high yields of styrene, benzene, toluene, xylene, and ethylbenzene, among others. Styrene has the highest yield, reaching up to 84.74% [70]. The application of PS pyrolytic oil as a fuel is questionable due to its high aromatic content, differentiating it from diesel; however, it has a similarity of about 60% with gasohol [125].

Furthermore, the mixture of PS with biomass in a co-pyrolysis process favors the liquid production that biomass by itself would not achieve and, in addition, improves its properties, such as increasing the heating value to values close to 41 MJ kg^−1^ and reducing up to 8.67% of its viscosity value [146,147]. It is concluded that the thermo-catalytic pyrolysis process is an effective recycling method to reduce a large amount of PS waste in the environment and convert it into high-value products for the chemical and petrochemical industry.

## Figures and Tables

**Table 1:** Summary of chemical properties of different PS types.

Properties	Virgin PS [26,52,65]	PS Waste [50,66,67,68]	EPS Waste [40,69,70]	HIPS Waste [40]
Ultimate analysis, wt.%				
C	91.93–92.60	87.40–92.20	91.69–92.34	66.47
H	7.40–8.00	7.51–9.43	6.93–8.28	7.63
N	0.00	0.00–0.64	0.11–0.51	0.14
S	0.04	0.08–0.15	0.00	0.00
O (by difference)	0.00	0.19–2.59	<0.22	17.84
Proximate analysis, wt.%				
Volatile matter	99.70–99.90	95.68–99.59	99.80	88.90
Fixed carbon	0.06–0.30	0.12–2.25	0.10	1.70
Ash content	0.01	0.17–1.77	0.02–0.10	7.60
Moisture	<0.10	0.24–0.30	0.00	1.80

**Table 2:** Summary of production of aromatic hydrocarbons from PS thermo-catalytic pyrolysis.

Aromatic	Catalyst	Reactor	Temperature [°C]	Heating Rate [°C min^−1^]	Residence Time [min]	Yield [%]	Ref.
***Thermal pyrolysis***							
Styrene	-	Batch	650	15	n.r.	84.74	[70]
Toluene	-	Small pilot scale	450	10	75	25.60	[111]
Ethylbenzene	-	Small pilot scale	450	10	75	21.20	[111]
α-Methyl styrene	-	Semi-batch	350	n.r.	n.r.	9.50	[102]
Benzene	-	CSBR ^1^	600	25	20	1.96	[122]
Cumene	-	Semi-batch	350	40	30	1.21	[104]
***Catalytic pyrolysis***							
Styrene	Zn/Al_2_O_3_	Pyrex	450	n.r.	120	62.88	[103]
α-Methyl styrene	Synthetic Zeolite	Small pilot scale	450	10	75	38.40	[111]
Ethylbenzene	FeCu/Al_2_O_3_	Batch	250	4	60	36.22	[18]
Benzene	Synthetic Zeolite	Small pilot scale	450	10	75	16.30	[111]
Toluene	15%Mg/Al_2_O_3_	Batch	600	25	120	13.10	[114]
Cumene	Synthetic Zeolite	Small pilot scale	450	10	75	8.10	[111]

n.r. = not reported. ^1^ Conical spouted bed reactor.

**Table 3:** Summary of fuel properties from PS pyrolysis and commercial fuels.

Fuel Properties	PS Pyrolysis Oil						Commercial Fuels		
	Temperatures [°C]						Gasoline [115,130,131,132]	Diesel [115,125,130,131,132]	Kerosene [115]
	150–379 [133]	400 ^1^ [115]	500 [125]	550 [67]	~695 [52]	~782 [52]			
Density, g cm^−3^	0.95	0.79	0.85	0.92	0.95	0.98	0.72–0.78	0.80–0.87	0.78–0.82
Kinematic viscosity, mm^2^ s^−1^	0.92	1.34	1.71	0.88	0.97	1.26	1.08–1.17	1.90–5.30	1.54–2.20
Flashpoint, °C			48	30.50	79	79	>42	>48	
Pour point, °C	<−35		19		−39	−39		−6 to 19	
Calorific value, MJ kg^−1^			43.55		40.89	40.02		46.95	

^1^ Mixed with CuO catalyst, 98:2.

**Table 4:** Energy content of co-pyrolysis of PS and biomass seeds.

Type	Seed: PS Ratio	HHV [MJ kg^−1^]	References
Grape seeds	100:0	35.49	[144]
	90:10	36.90	
	80:20	40.40	
	80:20	40.90 *	[145]
Coffee grounds	100:0	25.91	[148]
	75:25	26.68	
	50:50	33.98	
	25:75	39.66	
Rubber seeds	1:0	32.25	[147]
	2:1	37.48	
	1:1	37.61	
	1:2	41.10	
Karanja seeds	1:0	37.65	[149]
	1:1	38.88	[146]
	2:1	42.18	
	4:1	29.02	
	8:1	26.53	
Niger seed	1:0	35.87	[150]
	1:1	32.15	[146]
	2:1	41.42	
	4:1	31.82	
	8:1	29.77	

* LHV, MJ kg^−1^.

## Data Availability

Data sharing not applicable.
